# Who is eligible for lung cancer screening, and where are they? National subpopulation estimates of people eligible for lung cancer screening

**DOI:** 10.1002/cncr.70634

**Published:** 2026-10-09

**Authors:** David Haynes, Jihyoun Jeon, Gerard Silvestri, Christine Lambert, Rafael Meza, R. Adams Dudley, Anne Melzer, Arianne Baldomero, Anne Joseph, Abbie Begnaud

**Affiliations:** ^1^ Institute for Health Informatics University of Minnesota Minneapolis Minnesota USA; ^2^ School of Public Health University of Michigan Ann Arbor Michigan USA; ^3^ Department of Medicine Medical University of South Carolina Charleston South Carolina USA; ^4^ Department of Medicine, Division of Pulmonary, Allergy, Critical Care and Sleep Medicine University of Minnesota Minneapolis Minnesota USA; ^5^ Pulmonary, Allergy, Critical Care & Sleep Medicine, Veterans Administration Medical Center Minneapolis Minnesota USA

**Keywords:** lung cancer, cancer screening, small area estimates, health geography

## Abstract

**Background:**

The utilization of lung cancer screening services is less than 20%. There are a myriad of barriers at the patient, provider, and health care system levels that account for such low uptake. One reason lung cancer screening rates remain low is that identifying who is eligible is challenging at the patient and at the population level because a quantitative estimate of smoking history (i.e., 20 pack‐years of cigarette smoking exposure) is required to determine eligibility. This pack‐year determination is often missing from clinical documentation and is also absent in large health survey data.

**Methods:**

The Smoking History Generator was integrated into national cross‐sectional surveys to estimate smoking exposure intensity and duration for older US adults who have smoked. National estimates, at the substate level, for the number of individuals eligible for lung cancer screening are presented.

**Results:**

A publicly available dataset that enables researchers to estimate the population eligible for lung cancer screening at the census tract level was developed. Additionally, this approach permits exploration and description of the eligible population by age, sex, racial/ethnic groups, and current or former smoking status.

**Conclusion:**

The dataset can be aggregated by geographic location (e.g., county, state) and filtered by age range, sex, race/ethnicity, and smoking status to estimate specific subpopulations eligible for lung cancer screening. This dataset will greatly enhance future programmatic efforts to identify and direct resources to individuals eligible for lung cancer screening.

## Abstract 2

We developed a publicly available census tract‐level estimates the population eligible for lung cancer screening based on the 20 pack‐year history. The dataset supports will enable health professional and public health programs identify eligible populations and better target lung cancer screening resources.

## INTRODUCTION

In the United States, lung cancer remains the leading cause of cancer deaths.^1^ Fortunately, lung cancer screening (LCS) with annual low‐dose chest computed tomography reduces mortality through early detection.^2^, ^3^ However, LCS adoption has been slow, with about 17% of eligible individuals screened.^4^, ^5^, ^6^ There are many barriers to LCS, but one reason for low uptake is the difficulty determining who is eligible, which is determined by age and quantitative personal smoking exposure (i.e., how many packs of cigarettes are used daily and for how many years, or pack‐years). To increase LCS, a clinic may need to commit additional staff time to access smoking and perform patient education; therefore, it may be helpful for clinic leadership to know what the relative proportion of their patients are likely eligible for LCS.

There have been many different methods proposed to estimate LCS‐eligible population, as summarized in Table 1. Earlier studies had limitations related to a mismatch of data sources and eligibility criteria. For example, Triplett et al. use the previous United States Preventive Services Task Force (USPSTF) eligibility criteria (ages 55–80 and 30 pack‐years’ smoking history) that have since been expanded.^7^, ^8^, ^9^, ^10^, ^11^ In 2021, new USPSTF guidelines expanded LCS eligibility to individuals aged 50 to 80 years and lowered the smoking exposure threshold to 20 pack‐years. Naryan et al. used these new guidelines to examine the relationship between screening behavior and race/ethnicity.^12^ More recent estimates by Henderson et al. and Young et al. use the expanded USPSTF eligibility criteria and also provide estimates for people who continue to smoke (PWS) as well as people who formerly smoked (PWFS).^13^, ^14^ Henderson’s estimates integrated the 2022 Behavioral Risk Factor Surveillance System (BRFSS) data, which included detailed smoking history/exposure as a core component of the survey in each state. Their estimates are at both the national and state levels. Young et al. use the Life‐Years gained From Screening Computed Tomography prediction model to estimate individuals eligible for LCS. The model integrates three datasets: the National Health Interview Survey (NHIS), the Prostate, Lung, Colorectal, and Ovarian Cancer Screening Trial, and the National Lung Screening Trial.

**TABLE 1:** Comparison of published methods for estimating individuals eligible for lung cancer screening in the United States.

Author	Research Question	Year	Population	Data Sources	Methods	Findings
Young	LCS eligibility racial disparities	2025	50–80 years old ever smoked	NHIS (2015)	Researchers used the life‐years gained from the screening computed tomography prediction model. This model uses the NHIS and Cox models to estimate mortality among individuals who are ever smokers.	6915 NHIS participants aged 50 to 80 years who ever smoked represented 44 million individuals
Henderson	LCS uptake prevalence	2024	50–79 years old smoking (or quit in the past 15 years)	BRFSS (2022)	BRFSS + LCS section for all states. The specific statistical model is not mentioned.	16.4% of 13.5 million LCS‐eligible individuals are up to date
McIntire	Predicting LCS eligibility in Pennsylvania	2022	55–80 years old	NHIS (2015), Public Health Management Corporation Household Survey (2010,2012,2015)	Proportional fitting weight techniques were applied to match census demographic distributions to the model	Number of individuals eligible by planning district. Estimates are for both historic and current guidelines
Sahar	Who has geographic access to LCS facilities	2022	50–80 years old	ACS (2020)	The screening‐eligible population (SEP) = SEP (% eligible × state population), based on estimates from Fedawa et al. Distances from population centers to ACR screening facilities were calculated, and the number of individuals who had access within the increasing distances.	14 million LCS‐eligible persons; 5% of the eligible population did not have access to lung cancer screening facilities within 40 miles
Boudreau	Geographic access to lung cancer facilities for veterans	2021	Veterans (55–79 years old, smoked within 10 years)	BRFSS (2018)	Researchers estimate LCS‐eligible veterans for each state based on age and smoking history. They multiplied these proportions of LCS‐eligible veterans by each state’s veteran population to estimate the number of LCS‐eligible Veterans.	The geographic distribution of screening facilities and the number of veterans screened vary in proportion to state or regional need
Narayan	Association between LCS and race/ethnicity	2021	50–79 years old	BRFSS: 20 states (2019)	Researchers used cross‐sectional survey data to compare eligibility for old vs. new LCS. They used multivariate regression to determine the relationship between race/ethnicity and eligibility of LCS.	The revised USPSTF guidelines increased the population eligible for lung cancer screening.
Fadewa	State variation of LCS rates	2020	55–80 years old 2013 USPSTF	NHIS (2015), BRFSS (2016–2018), BRFSS, US Census Bureau	Numerator: LDCT reported to ACR LCSR Denominator: Estimated screening eligible population = SEP (% eligible × state population)	US 5% LCS eligible ever screened
Triplette	Predicting LCS eligibility	2019	55–77 years old ever smoked	NHANES (2011–2016)	Determine the predictive value of simplified criteria for actual LCS eligibility	Age and smoking status are highly predictive for LCS eligibility

Abbreviations: ACS, Annual Community Survey; BRFSS, Behavioral Risk Factor Surveillance System; LCS, lung cancer screening; NHANES, National Health and Nutrition Examination Survey; NHIS, National Health Interview Survey.

The studies in Table 1 have contributed an important understanding of how many people in the United States might benefit from LCS using administrative datasets. However, all of those estimates are at the national and state levels. Health systems would be better positioned to evaluate investing in LCS if they had a local and granular understanding the size of the patient population eligible for LCS. This would require creating estimates of small areas that accounted for patient demographic subpopulations.

The objective of this study is to estimate the LCS eligible population of the United States with small‐area and demographic specificity by improving upon previously published estimates using additional methods. We present a new approach that estimates LCS‐eligible individuals by subpopulations and for small areas. We combined demographic information from the American Community Survey (ACS), BRFSS data, and birth cohort smoking pack‐year distributions from the Smoking History Generator (SHG) developed by the Cancer Intervention and Surveillance Modeling Network ‐ Lung Working Group (CISNET‐LWG) to develop these estimates.^15^ This study will integrate smoking pack‐years and intensity estimates to help quantify the number of persons eligible for LCS. The main contribution of this work is the refinement of existing estimates with additional demographic and geographic specificity, but these estimates could also be used to optimize and measure the efficiency and reach of LCS services and smoking cessation resources.

## METHODS

### Datasets

We integrated three data sources: the 2016–2020 BRFSS to estimate smoking prevalence, the 2016–2020 ACS to obtain census tract demographic counts, and the CISNET‐LWG SHG to estimate pack‐year smoking histories.

#### Behavioral Risk Factor Surveillance System

The BRFSS is a nationally representative dataset commonly used to examine population‐level behaviors among PWS and PWFS.^16^ We combined the 2016 to 2020 datasets to create the prevalence estimates of PWS and PWFS for each state in the United States.

#### American Community Survey

The ACS provides population‐level demographic data for the United States at the census tract level. These data contain five‐year population estimates for subpopulations (e.g., by age, sex, race/ethnicity) downloaded from the National Historical Geographic Information System.^17^

#### Smoking History Generator

The CISNET‐LWG SHG integrates data from the NHIS to create models of birth cohort–specific, individual‐level smoking history for age, sex, and race/ethnicity combinations.^15^, ^18^ The SHG has been used to understand disparities in lung cancer screening guidelines^19^ and model the effectiveness of tobacco control policies,^20^, ^21^ evaluate the benefits and harms of various lung cancer screening strategies, estimate the effectiveness and cost‐effectiveness of LCS programs, and evaluate the effect of smoking cessation in LCS settings.^22^, ^23^, ^24^, ^25^ We use the smoking history data simulated by the SHG to assign smoking exposure estimates in pack‐years to PWS and PWFS.

### Data integration

Integrating data from diverse sources is a complex process. Therefore, to improve reproducibility, we briefly describe the tools and processes used for integrating the data. We also provide additional documentation of our data transformation pipeline and provide access to our public repository, which contains the code. Data preprocessing and harmonization were performed using automated scripts in Python 3.7.

#### BRFSS data harmonization

The BRFSS is a national survey, and each state receives funding to administer the required core modules, which focus on demographics and health behaviors, as well as the tobacco module. The tobacco cessation module comprises five questions used to estimate the prevalence of current and former smokers.^16^ We developed a harmonization script that aligned the smoking module questions and demographic categories, given variations in the data fields. Once the data were harmonized, smoking prevalence and smoking cessation data were calculated.

### Creating cross‐sectional subpopulation prevalence rates

A cross‐sectional subpopulation is a specific demographic segment of the population (e.g., Black men aged 55–64). We will use this as an example of a cross‐sectional subpopulation, bearing in mind that there are various combinations from the eight age groupings, six racial/ethnicities, and two sexes.

We determined current and former smoking prevalence rates at the state level by integrating the harmonized 2016–2020 BRFSS survey response data describing smoking behaviors with the original BRFSS weights. We used the “_RFSMOK3” (current) and “_SMOKER3” (former) variables to create current and former smoking prevalence rates by age, sex, and race/ethnicity. Individuals were classified as current smokers if they have smoked at least 100 cigarettes in their lifetime and smoke every day or smoke some days. We observed that direct cross‐sectional subpopulation estimates were unstable because of small cell sizes. Therefore, we assumed that the average of these larger demographic categories would approximate what was observed in the cross‐sectional populations. Therefore, the rate for current Black males aged 55–64 is the average of the following rates: Black people, males, and people aged 55–64.

Former smoking prevalence rates were determined to estimate PWFS. The BFRFSS initially defines PWFS using the “_RFSSMOK3” variable as people who have smoked at least 100 cigarettes in their lifetime and currently do not smoke. We included the BRFSS variable “LASTSMK2” to calculate former smoking rates. The LASTSMK2 variable response options range from “less than 1 year” to “more than 10 years” ago. To calculate the rate of PWFS eligible for lung cancer screening, we multiplied the former smoking rate by the reciprocal of the quit rate, thereby identifying LCS‐eligible PWFS as individuals who report having quit smoking, but not more than 10 years ago.

### Integrating census data

Once the smoking and cessation rates were determined, it was integrated with the census tract‐level demographic data to predict the number of individuals who are eligible for LCS by location. We used five large census racial categories for our analysis (i.e., American Indian or Alaska Native, Black, Pacific Islander, and White) and one ethnic grouping, Hispanic. For each race/ethnicity, there are 20 categories describing populations aged 18 and older by sex. Eight of these age categories (i.e., 45–54, 55–64, 65–74, 75–84) were relevant. This results in 40 age‐sex‐race/ethnicity combinations, which are aligned to BRFSS smoking prevalence categories. The census age‐sex‐race/ethnicity combinations represent the number of individuals that reside in this area for each combination of demographic characteristics (e.g., census data reports, for each census tract, the number of Black males aged 55–64). We use this information to estimate the number of current and former smokers at the census tract level.

Unlike the BRFSS, census age distributions typically use 10‐year age groups (e.g., 55–64). However, current eligibility criteria for lung cancer screening apply only to individuals aged 50 to 80 years. To account for screening eligibility criteria, we created new age groups and evenly distributed the population across them (e.g., the 45–54 age group was divided equally into 45–49 and 50–54). Each new sub‐age group received 50% of the current and former smoking populations in the original range. This technique was applied to the 75 to 84 age group, with the 75 to 80 age group receiving 60% of the population and the 81 to 84 age group receiving 40%.

### Integrating Smoking History Generator

The CISNET‐LWG SHG models total pack‐year histories for PWS and PWFS, which can be assigned to individuals to estimate their eligibility for lung cancer screening. A limitation of the SHG is that it provides a single combined estimate for two large racial groups: Asians and Pacific Islanders, which are distinct racial identities in the census. We use the same estimate for each group.

Despite this limitation, the SHG provides distributions (e.g., mean, SD) of total pack‐year estimates for PWS and PWFS by cross‐sectional populations, which is used to simulate behavior. To estimate smoking pack‐years for PWS and PWFS, we assume a normal distribution and assign the mean and SD of a cross‐sectional population (e.g., Black males aged 55–64). We then randomly sampled from this distribution according to the number of individuals residing in each census tract. Therefore, if there are 30 Black males aged 55 to 64 years living in a census tract, we will draw 30 times from that distribution. Each individual will be assigned a probability that can be compared against the 20 pack‐year smoking threshold value. If the value is at or above the threshold, the individual is eligible for lung cancer screening. We then simulate this draw‐and‐sample approach 500 times. The result is a distribution for each cross‐sectional population and each geographic unit.

### Storing and visualizing lung cancer screening eligible data

Once all census tracts have been estimated, the data are loaded into a database. PostgreSQL was used to store the data describing the individuals eligible for LCS. The data were loaded into a denormalized structure to facilitate analysis and visualization based on LCS eligibility criteria and populations of interest. We used ArcPro 3.0.3 to query the database and create maps describing national and state LCS‐eligible populations. The data are available for download at https://conservancy.umn.edu/items/86b51a54‐8ff9‐4235‐9bb4‐5aa0b1ad408f.

## Results

We estimate that there are approximately 26.4 million people aged 50 to 80 years with at least a 20 pack‐year smoking history in the United States. More than half of LCS‐eligible persons, or 16.1 million, continue to smoke cigarettes (see Table 1), whereas 10.3 million used to smoke (Table 2). Table 2 provides a detailed description of the racial and ethnic groups eligible for LCS according to the 2021 USPSTF guideline. White Americans are the largest racial/ethnic group eligible for LCS at 19.6 million. However, American Indians and Alaska Natives are the racial group with the highest proportion of eligible persons at 34.21% of the population aged 50 to 80 years being eligible, whereas about one quarter of White and Black Americans aged 50 to 80 years are expected to be eligible for LCS. Adults 55 to 64 years old are the largest LCS eligible age group, with most of those still smoking (6.9 million) and 4.1 million having quit smoking. National Summary statistics of the simulation results are provided in Supporting Information S1: Table A1 and state‐level estimates are provided in Supporting Information S1: Table A2. Supporting Information S1: Table A3 reports outliers found in the estimates. There are 25 (0.02%) geographic census tracts of 84,121 where our estimates outnumber the population. These features had very small populations, with counts of people aged 50 to 80 years ranging from 1 to 9.

**TABLE 2:** National estimates for PWS and PWFS based on the 2020 census.

	PWS (Mean)	PWS (SD)	PWFS (Mean)	PWFS (SD)	Mean PWS and PWFS aged 50–80	Percent of population aged 50–80	US Population aged 50–80
Total eligible	16,104,508		10,333,219		26,437,727		
American Indian	148,779.0	1.4	96,277.1	0.82	245,056.13	34.21%	716,358.50
Asian	607,655.0	2.4	471,687.6	1.86	1,079,342.64	20.99%	5,143,122.60
Black	1,829,046.2	6.4	1,025,218.1	3.32	2,854,264.38	25.34%	11,265,239.90
Hispanic	1,499,926.0	4.8	1,096,913.6	3.41	2,596,839.59	22.15%	11,725,611.80
Pacific Islander	23,584.1	0.4	19,078.6	0.33	42,662.67	29.53%	144,456.50
White	11,995,517.8	17.4	7,624,044.4	10.33	19,619,562.28	24.82%	79,046,742.70
50–54	3,527,097.2	7.2	2,147,599.7	4.20	5,674,696.92	25.29%	22,439,342.00
55–64	6,990,274.9	15.1	4,195,996.9	8.74	11,186,271.81	25.22%	44,349,836.00
65–74	4,231,757.6	10.0	3,006,383.5	6.81	7,238,141.08	22.79%	31,754,102.00
75–80	1,355,378.5	3.5	983,239.4	2.45	2,338,617.88	24.62%	9,498,252.00
Female	8,065,497.4	10.1	5,119,844.8	6.05	13,185,342.14	23.31%	56,576,888.80
Male	8,039,010.8	10.1	5,213,374.7	6.26	13,252,385.55	25.75%	51,464,643.20

Abbreviations: PWFS, people who formerly smoke; PWS, people who continue to smoke.

In addition to providing updated estimates for racial/ethnic groups, our approach provides these estimates at census tract level, which improves the spatial resolution compared to prior studies (Table 1). Figure 1 shows the total number of people eligible for LCS by census tract, grouped by quintiles, whereas Figure 1A shows the same data, focusing on minority populations (e.g., American Indian, Asian, Black, Hispanic, Pacific Islander). Figure 1A,B use the same legend for easy comparison. Figure 1B shows that the largest populations are found in many rural and remote areas that correspond to Tribal Nations. Additionally, the Southeastern region of the United States has many census tracts with large numbers of eligible African Americans.

**FIGURE 1:**
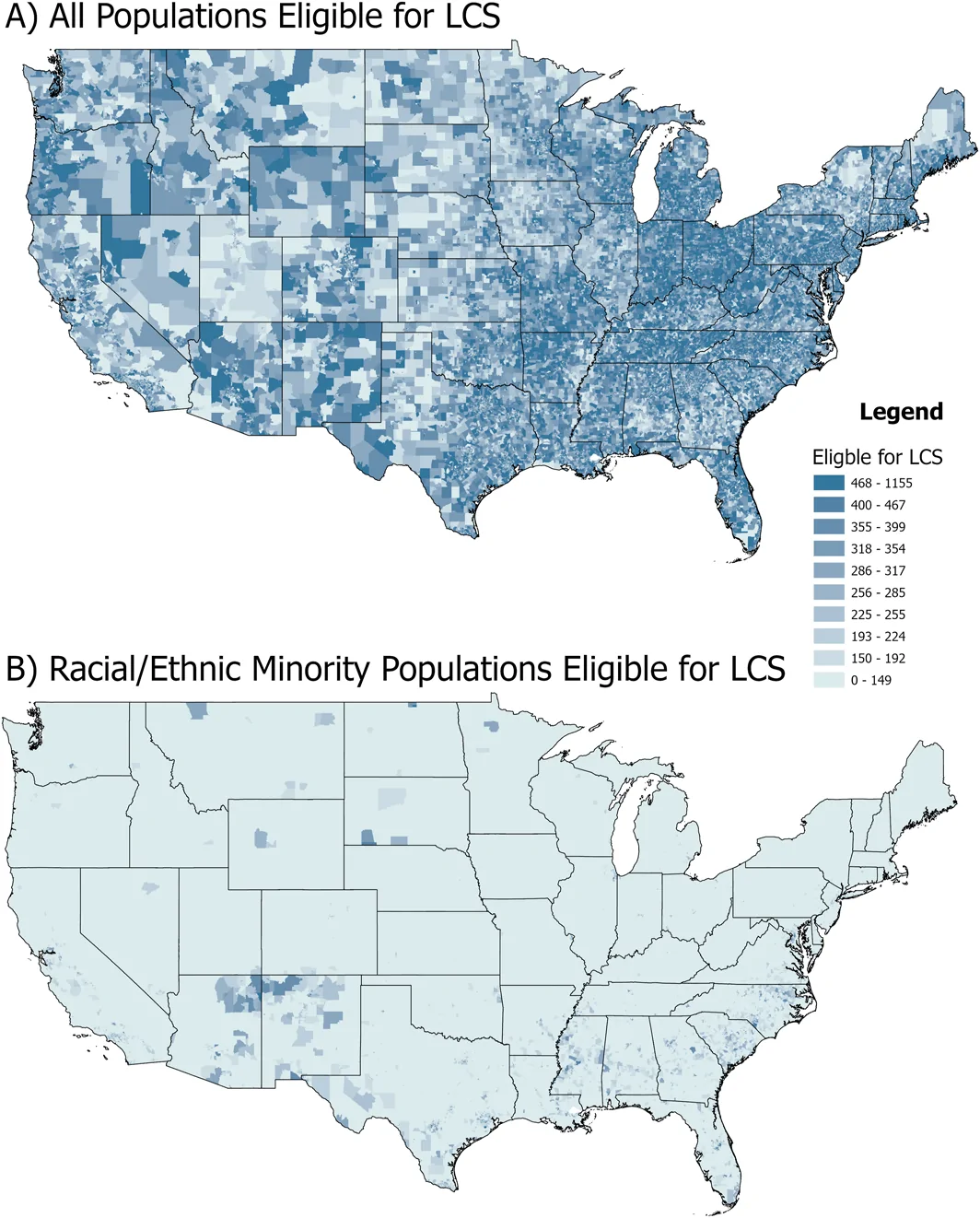
2020 census tract estimates of total and percentage of persons eligible for lung cancer screening.

The estimates of individuals eligible for LCS are made at the census tract level for age‐race‐sex populations. Supporting Information S1: Table A4 provides national estimates for all subpopulations. This means that different priority populations can be chosen and aggregated to make new maps that support different health system or public health planning efforts. Figure 2 provides exemplar county‐level maps. Figure 2 depicts two different age‐race populations that have been chosen because of their high rates of lung cancer incidence and current smoking prevalence. Each map’s legend is unique, highlighting the wide variation and differences in population magnitude. The maps should only be compared to each other if the cross‐populations are the same. The trends identified in these datasets are a sample of the many possibilities they enable.

**FIGURE 2:**
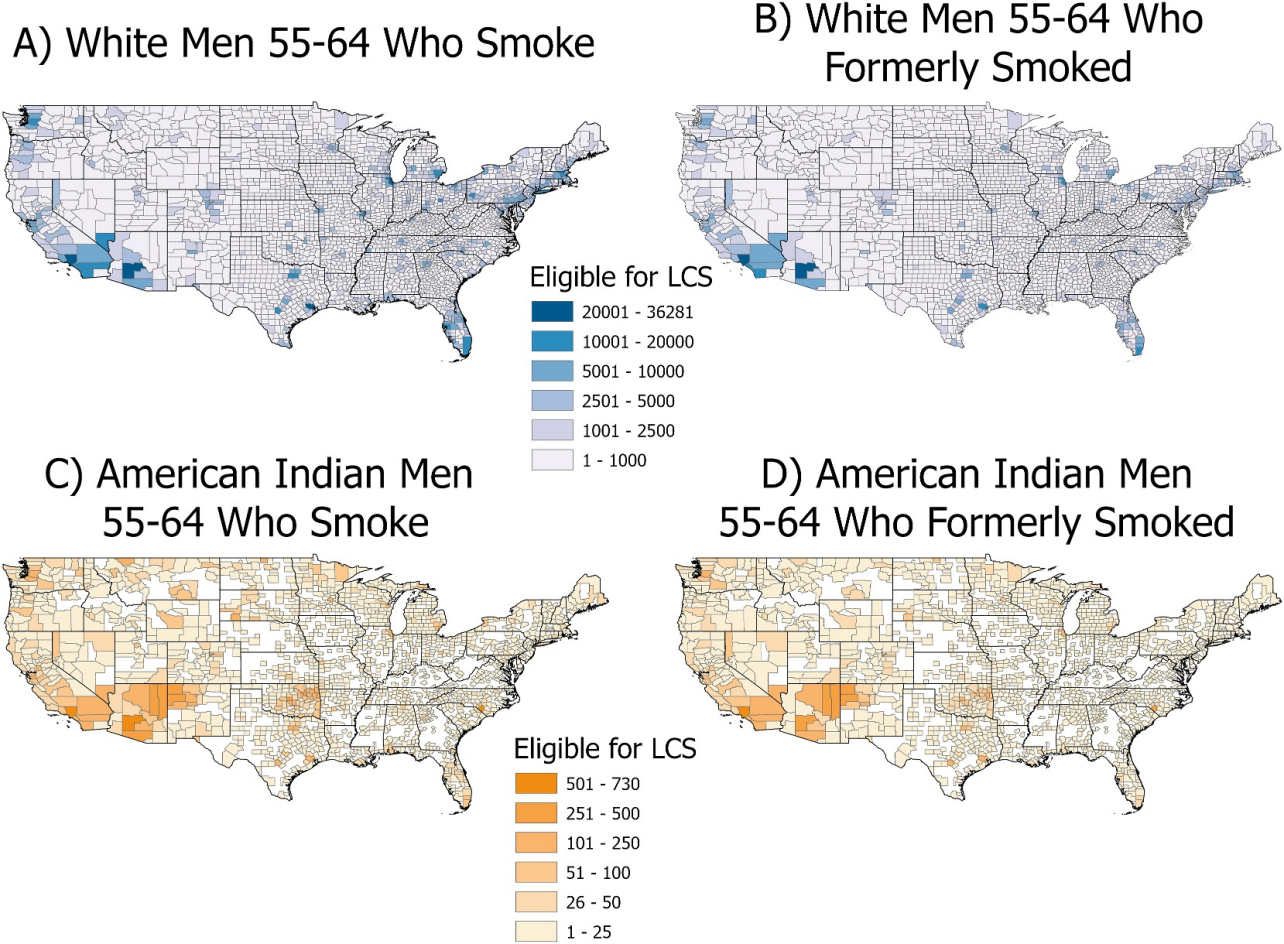
Largest and priority populations by county of those who are LCS eligible. LCS indicates lung cancer screening.

Figure 2A displays the population distribution of White males, aged 55 to 64 who currently smoke and are eligible for LCS and Figure 2B White males, aged 55 to 64, who quit. Historically, White males have represented the largest smoking population (Table 2), but this population’s smoking behavior has decreased significantly.^26^ White males are the largest population in this dataset, and Figure 2A reflects this, with almost all major cities being identified as areas with a high number of White males who smoke being eligible.

The smoking prevalence for White males peaked for generations born between 1915 and 1925. Historical smoking population prevalence is a likely predictor of lung cancer occurrence.^27^ In contrast to Figure 2A,B has fewer population centers identified as areas with large numbers of PWFS. Instead, the counties that have the most PWFS eligible reside in the west coast (Washington, Oregon, and California), the Southwest (Arizona) and Northeast regions (Pennsylvania, Maryland, New Jersey, Washington DC, New York, and Massachusetts), and Florida.

Figure 2C and Figure 2D illustrate the geographic distribution of American Indian and Alaska Native males who currently smoke and those who have quit, aged 50 to 64, and are eligible for LCS. The spatial patterns of the American Indian and Alaska Native populations are also spatially distinct, with a higher concentration of individuals eligible for LCS residing in the Southwest region of the United States (i.e., Nevada, Arizona, New Mexico) and the rural areas of Montana and North and South Dakota.

It is important to consider the total number of individuals each group represents when interpreting the maps. In Figure 2A, White males who smoke and are aged 55 to 64, have the largest geographic distribution and the largest total population, with multiple counties having over 20,000 people eligible. In contrast, Figure 2C, American Indian and Alaska Native males aged 55 to 64 largest category is 500. This highlights the importance of separating data by race/ethnicity and smoking status to better understand and serve different populations.

## Discussion

This work fills a critical gap in the literature by providing enhanced estimates of PWS and PWFS eligible for LCS using the current 20‐year pack history. The estimates are enhanced in two ways. First, these are the only national estimates at the census tract level. Second, we provide these estimates for specific populations (i.e., White males aged 55–60 who currently smoke). These estimates can be used by health organizations or public health groups to plan, allocate, and market LCS resources and evaluate the efficacy of interventions or community‐based programs.

This work builds upon the work of many different researchers (Table 1). Triplette, Fedewa, and Sahar created some of the first national estimates for those eligible for LCS. In general, our work builds on these approaches using BRFSS and census data, but we introduce the SHG to create our estimates. The most recent estimates by Henderson and Young are the most similar to our proposed estimates. Henderson’s estimates leverage the BRFSS and are similar to Fedewa’s approach, whereas Young uses the 2015 NHIS and statistical models to create the estimates.

Table 3 compares our proposed estimates with those of Henderson and Young. Overall, our estimate of 26.4 million people is reasonable, falling between the Henderson and Young estimates of 13.5 and 44 million, respectively. We find similar results between the proposed and Henderson estimates for the racial/ethnic groups American Indian and Pacific Islander. The proposed estimates differ most strongly from Henderson's estimates among Asian, Black, and Hispanic groups. However, the proposed estimates align with Young's estimates on those groups.

**TABLE 3:** Comparison of estimates of total persons eligible for LCS to the percentage of the US population 50–80.

	Proposed	Henderson	Young
American Indian	245,022 (34.20%)	308,728 (43.10%)	‐‐‐
Asian	1,079,297 (20.99%)	175,228 (3.41%)	1,266,936 (24.63%)
Black	2,854,183 (25.34%)	1,138,680 (10.11%)	4,333,903 (38.47%)
Hispanic	2,597,005 (22.15%)	641,116 (5.47%)	3,569,321 (30.44%)
Pacific Islander	42,671 (29.54%)	43,064 (29.81%)	‐‐‐
White	19,619,670 (24.82%)	10,617,578 (13.43%)	34,868,520 (44.11%)
(Multiracial)	‐‐‐‐‐‐	47,056	‐‐‐‐‐‐
Missing	‐‐‐‐‐‐	554,898	‐‐‐‐‐‐
50–54	5,674,730 (25.29%)	2,063,840 (9.20%)	9,021,667 (40.20%)
55–64	11,186,281 (25.22%)	5,977,366 (13.48%)	17,316,076 (39.04%)
65–74	7,238,255 (22.79%)	4,386,720 (13.81%)	13,406,633 (42.22%)
75–80	2,338,583 (24.62%)	1,098,421 (11.56%)	4,294,304 (45.21%)
Female	13,185,323 (23.31%)	6,177,439 (10.92%)	20,554,860 (36.33%)
Male	13,252,526 (25.75%)	7,348,909 (14.28%)	23,483,820 (45.63%)
Total	26,437,849 (24.47%)	13,526,348.00 (12.52%)	44,038,679.00 (40.76%)

Abbreviations: LCS, lung cancer screening.

Across the age groups, there is no consensus among the three estimates. Our proposed estimates are based on U.S. Census population data, whereas the others model the population using BRFSS and National Institutes of Health datasets. The second discrepancy we identify is with the female‐to‐male ratio. The proposed estimates only suggest a 2% difference between the groups, which is comparable to Henderson (3.3%). However, Young's estimates suggest a wider discrepancy at about 9%.

Although work should continue to refine and improve the estimates, it is very clear that there are large disparities in cancer screening rates in the U.S. The disparities vary by race and ethnicity, for the uninsured and underinsured populations,^26^, ^27^, ^28^, ^29^ and in rural populations.^30^ Because LCS requires a smoking history for screening eligibility, estimates are important for addressing potential disparities by race and ethnicity. This is especially important for populations with a high prevalence of smoking (e.g., American Indians, Alaska Natives).

### Clinical considerations

Our approach was to provide estimates at a small enough geographical level that they would support maximum flexibility for real‐world application. Figure 2 demonstrates this by providing LCS estimates at the county level, but few clinics or even entire health systems serve entire counties. Health systems often have multiple clinical locations, and providing the data at the census tract level is advantageous for different potential use cases (e.g., health systems and those planning clinical interventions could benefit from LCS estimates at smaller geographical units). Additionally, it is common for individual clinics to serve patient populations of certain racial/ethnic groups, so the census tract‐level data stratified by demographic subpopulations could improve planning and resource allocation.

Potential applications of this work include determining the location of new service locations and the capacity for LCS services, including low‐dose computed tomography and smoking cessation resources. This can be done by identifying the census tracts in which patients reside to determine the patient population necessary to implement services. A public‐private partnership with public health departments and clinical service providers like hospitals and clinics, to ensure that adequate imaging capacity exists. Where existing imaging capacity is not sufficient, efforts could be made to enhance the capacity (e.g., with extended hours or the use of mobile units).

### Limitations

This dataset provides population‐level estimates but does not replace the need for clinician to determine an individual patient’s eligibility for LCS. An important limitation of these estimates is that they use the BRFSS for estimating smoking prevalence. BRFSS rates tend to be more stable for PWS than for PWFS. BRFSS rates are unstable for subpopulations because of the small number of respondents. Supporting Information S1: Figure A1 provides illustrations of the distribution of smoking prevalence rates for PWS and PWFS by racial/ethnic categories over a five‐year period. Instability increases as we reduce the population from large racial ethnic groups and subdivide it by sex and/or age groups, even when the BRFSS dataset was extended to 10 years.

We used the SHG to generate smoking pack‐years and smoking intensity at the national level. This resulted in a few census tracts (25, 0.02%) being estimated to have more PWS than persons residing in those tracts. Additional measures (e.g., minimum, mean, maximum, SD) can be found in Supporting Information S1: Tables A1 and A2, which could provide guidance on the number of individuals likely to meet the clinical definition of eligible for LCS. Another important limitation is that, although our methods expand understanding of LCS‐eligible PWFS, these estimates assume a remote quit time that does not perfectly align with the 2021 USPSTF guidelines. We use a 10‐year quit time to estimate LCS‐eligible PWFS. We believe that this is a conservative estimate of the total number of PWFS eligible for LCS (e.g., a person who quit smoking 12 years ago would be eligible for LCS by USPSTF but be considered ineligible in our estimates). Therefore, we are likely undercounting the number of people eligible for LCS.

However, more recently, the American Cancer Society has endorsed LCS for individuals aged 50 to 80 years with a 20 pack‐year history, irrespective of quit time. For a sensitivity test, we conducted this analysis using the BRFSS definition of former smoker, “who reported having smoked at least 100 cigarettes in their lifetime and currently do not smoke.” This resulted in approximately 26 million people being eligible for lung cancer screening. This approach could be useful for estimating the number of people eligible for LCS according to the American Cancer Society recommendations.

Last, the BRFSS and SHG use different racial/ethnic categories (i.e., Black_alone or non‐Hispanic Black). These population definitions are not perfectly aligned, which could lead to different estimates. However, the census does not provide any population estimates at the subpopulation level at this time.

### Conclusions

This work provides updated national estimates of lung cancer screening eligible populations that align with current guidelines. This work has integrated a new dataset, which enables estimation of smoking intensity and total pack‐years. This work provides a new dataset with increased spatial resolution that can be used to implement and coordinate resources for LCS interventions. Specific applications of this work include planning service locations and the capacity of LCS services, including low‐dose computed tomography and smoking cessation resources. Other efforts could use these data to identify geographic areas or particular populations to direct marketing and resources about LCS services. Last, the dataset provides a useful foundation for estimating the uptake of LCS screening services at the national and local levels.

## AUTHOR CONTRIBUTIONS

**David Haynes:** Conceptualization; investigation; writing—original draft; methodology; validation; visualization; funding acquisition; writing—review and editing; software; formal analysis; data curation. **Jihyoun Jeon:** Methodology and software. **Gerard Silvestri:** Supervision; writing—review and editing. **Christine Lambert:** Writing—review and editing. **Rafael Meza:** Methodology; software. **R. Adams Dudley:** Methodology; writing—review and editing; supervision. **Anne Melzer:** Writing—review and editing. **Arrianne Baldomero:** Writing—review and editing. **Anne Joseph:** Supervision; funding acquisition; conceptualization. **Abbie Begnaud:** Writing—review and editing; writing—original draft.

## CONFLICT OF INTEREST STATEMENT

The authors declare that they have no known competing financial interests or personal relationships that could have influenced the work reported in this manuscript.

## Supporting information

Supporting Information S1

## Data Availability

The data that support the findings of this study are openly available in Data Repository for the University of Minnesota at https://conservancy.umn.edu/items/86b51a54‐8ff9‐4235‐9bb4‐5aa0b1ad408f.
