# Supplementary material for: Who is eligible for lung cancer screening, and where are they? National subpopulation estimates of people eligible for lung cancer screening
Source: Cancer. 2026 Oct 9;132(20):e70634. doi: 10.1002/cncr.70634 (PMC13650490; doi:10.1002/cncr.70634)
Supplement: Supplementary file 1 [file CNCR-132-e70634-s001.docx]

Appendix

Table A1 - Summary Statistics for LCS Simulations

Table A2 - State Estimates of PWS and PWFS

Table A3 - LCS Estimates Outliers: Census Tracts Where Estimates of LCS eligible persons Outnumber the Total Population

Table A4 - Subpopulation Estimates and Percentages of PWS and PWFS

Figure A1 - Distribution of BRFSS Prevalence Rates for Current Smoking and Quit Smoking, 2016-2020.

Table A1. Summary Statistics for LCS Simulations

|  | **Currently Smoking** | | | | **Formerly Smoked** | | | | **Total US Population** |
| --- | --- | --- | --- | --- | --- | --- | --- | --- | --- |
|  | *(Min)* | *(Mean)* | *(Max)* | *(StDev)* | *(Min)* | *(Mean)* | *(Max)* | *(StDev)* |  |
| American Indian | 76,504.0 | 148,776.2 | 161,473.0 | 1.4 | 28,879.0 | 96,246.0 | 111,466.0 | 0.82 | 716,358.50 |
| Asian | 322,037.0 | 607,653.4 | 661,503.0 | 2.4 | 167,938.0 | 471,644.0 | 567,681.0 | 1.86 | 5,143,122.60 |
| Black | 1,273,575.0 | 1,829,002.8 | 1,986,026.0 | 6.4 | 506,028.0 | 1,025,180.2 | 1,192,386.0 | 3.32 | 11,265,239.90 |
| Hispanic | 896,478.0 | 1,499,929.8 | 1,696,571.0 | 4.8 | 508,872.0 | 1,097,075.3 | 1,331,270.0 | 3.41 | 11,725,611.80 |
| Pacific Islander | 10,500.0 | 23,583.5 | 25,693.0 | 0.4 | 5,190.0 | 19,087.5 | 23,041.0 | 0.33 | 144,456.50 |
| White | 10,325,660.0 | 11,995,610.3 | 12,558,254.0 | 17.4 | 5,595,040.0 | 7,624,060.1 | 8,608,380.0 | 10.34 | 79,046,742.70 |
|  |  |  |  |  |  |  |  |  |  |
|  | **Currently Smoking** | | | | **Formerly Smoked** | | | | **Total Population** |
|  | *(Min)* | *(Mean)* | *(Max)* | *(StDev)* | *(Min)* | *(Mean)* | *(Max)* | *(StDev)* |  |
| 50-54 | 2,570,179.0 | 3,527,073.9 | 3,834,401.0 | 7.2 | 1,198,726.0 | 2,147,655.7 | 2,549,533.0 | 4.20 | 22,439,342.00 |
| 55-64 | 5,859,795.0 | 6,990,209.8 | 7,435,961.0 | 15.1 | 3,000,625.0 | 4,196,071.4 | 4,803,828.0 | 8.74 | 44,349,836.00 |
| 65-74 | 3,493,758.0 | 4,231,889.6 | 4,418,089.0 | 10.0 | 2,099,014.0 | 3,006,365.6 | 3,384,804.0 | 6.81 | 31,754,102.00 |
| 75-80 | 981,022.0 | 1,355,382.6 | 1,401,069.0 | 3.5 | 513,582.0 | 983,200.3 | 1,096,059.0 | 2.45 | 9,498,252.00 |
|  |  |  |  |  |  |  |  |  |  |
|  | **Currently Smoking** | | | | **Formerly Smoked** | | | | **Total Population** |
|  | *(Min)* | *(Mean)* | *(Max)* | *(StDev)* | *(Min)* | *(Mean)* | *(Max)* | *(StDev)* |  |
| Female | 6,455,211.0 | 8,065,565.0 | 8,552,819.0 | 10.1 | 3,343,644.0 | 5,119,757.9 | 5,878,479.0 | 6.05 | 56,576,888.80 |
| Male | 6,449,543.0 | 8,038,990.9 | 8,536,701.0 | 10.1 | 3,468,303.0 | 5,213,535.1 | 5,955,745.0 | 6.26 | 51,464,643.20 |

Table A2. State Estimates of PWS and PWFS

|  | **PWS** | | | | **PWFS** | | | |
| --- | --- | --- | --- | --- | --- | --- | --- | --- |
|  | *(Min)* | *(Mean)* | *(Max)* | *(StDev)* | *(Min)* | *(Mean)* | *(Max)* | *(StDev)* |
| AK | 28,701.0 | 36,405.2 | 38,646.0 | 10.5 | 15,058.0 | 23,480.1 | 26,890.0 | 6.5 |
| AL | 249,051.0 | 301,319.5 | 318,773.0 | 11.1 | 107,365.0 | 161,161.3 | 183,686.0 | 5.8 |
| AR | 163,650.0 | 194,091.7 | 204,780.0 | 12.6 | 65,875.0 | 96,683.1 | 110,085.0 | 6.1 |
| AZ | 276,492.0 | 349,191.2 | 371,447.0 | 10.1 | 167,213.0 | 249,471.0 | 285,607.0 | 7.1 |
| CA | 926,206.0 | 1,317,132.3 | 1,416,443.0 | 6.0 | 700,392.0 | 1,170,349.9 | 1,358,729.0 | 5.3 |
| CO | 186,896.0 | 238,710.4 | 253,499.0 | 8.5 | 122,767.0 | 183,258.8 | 209,698.0 | 6.5 |
| CT | 116,267.0 | 149,059.5 | 158,133.0 | 8.6 | 72,154.0 | 109,910.0 | 125,788.0 | 6.2 |
| DC | 18,641.0 | 25,413.5 | 27,361.0 | 6.7 | 9,454.0 | 16,415.6 | 18,974.0 | 4.0 |
| DE | 43,133.0 | 53,659.0 | 56,789.0 | 10.1 | 22,277.0 | 33,659.1 | 38,394.0 | 6.3 |
| FL | 1,006,785.0 | 1,235,997.1 | 1,314,474.0 | 11.9 | 574,756.0 | 828,562.5 | 947,083.0 | 7.9 |
| GA | 405,912.0 | 510,171.9 | 542,446.0 | 9.2 | 186,019.0 | 294,520.1 | 337,692.0 | 5.2 |
| HI | 31,572.0 | 47,901.6 | 51,506.0 | 4.5 | 18,566.0 | 36,942.9 | 43,366.0 | 3.5 |
| IA | 130,494.0 | 156,696.0 | 164,787.0 | 9.7 | 63,862.0 | 93,007.4 | 105,709.0 | 5.6 |
| ID | 61,444.0 | 74,916.4 | 78,953.0 | 9.1 | 34,568.0 | 50,198.4 | 57,145.0 | 5.9 |
| IL | 471,139.0 | 592,832.5 | 629,444.0 | 9.3 | 240,459.0 | 372,382.4 | 426,951.0 | 5.7 |
| IN | 351,749.0 | 411,583.8 | 433,594.0 | 13.7 | 148,754.0 | 210,681.1 | 239,367.0 | 6.8 |
| KS | 117,390.0 | 145,378.2 | 153,566.0 | 9.6 | 59,476.0 | 89,829.4 | 102,432.0 | 5.8 |
| KY | 274,803.0 | 318,803.4 | 335,220.0 | 13.8 | 112,971.0 | 158,527.6 | 179,805.0 | 6.7 |
| LA | 238,473.0 | 290,933.3 | 308,490.0 | 11.4 | 99,699.0 | 152,117.9 | 173,620.0 | 5.8 |
| MA | 229,099.0 | 285,737.1 | 302,183.0 | 9.6 | 129,441.0 | 193,250.0 | 220,606.0 | 6.3 |
| MD | 185,162.0 | 242,017.5 | 257,736.0 | 8.0 | 100,053.0 | 162,045.9 | 186,165.0 | 5.2 |
| ME | 71,501.0 | 82,914.8 | 86,934.0 | 12.1 | 38,235.0 | 51,441.3 | 58,116.0 | 7.4 |
| MI | 502,136.0 | 603,975.5 | 637,536.0 | 11.3 | 235,170.0 | 342,654.4 | 389,822.0 | 6.3 |
| MN | 199,204.0 | 245,988.7 | 259,139.0 | 8.9 | 117,045.0 | 170,948.8 | 194,408.0 | 6.1 |
| MO | 314,540.0 | 371,698.7 | 391,614.0 | 12.5 | 141,844.0 | 201,882.5 | 229,229.0 | 6.6 |
| MS | 155,134.0 | 188,094.7 | 199,494.0 | 10.8 | 53,794.0 | 85,181.3 | 97,380.0 | 4.8 |
| MT | 48,471.0 | 57,896.0 | 60,874.0 | 10.4 | 25,747.0 | 36,366.5 | 41,196.0 | 6.5 |
| NC | 461,404.0 | 566,837.7 | 600,312.0 | 10.8 | 219,683.0 | 330,778.5 | 377,662.0 | 6.2 |
| ND | 31,040.0 | 37,353.2 | 39,278.0 | 9.6 | 14,824.0 | 21,751.7 | 24,693.0 | 5.4 |
| NE | 69,780.0 | 86,614.9 | 91,383.0 | 8.4 | 37,762.0 | 56,740.5 | 64,701.0 | 5.4 |
| NH | 60,122.0 | 70,509.9 | 74,063.0 | 11.7 | 34,093.0 | 46,560.1 | 52,744.0 | 7.5 |
| NJ | 304,711.0 | 395,758.6 | 421,749.0 | 8.7 | 180,431.0 | 283,287.2 | 326,009.0 | 6.0 |
| NM | 99,628.0 | 127,042.9 | 136,332.0 | 9.4 | 53,227.0 | 82,442.3 | 95,290.0 | 5.9 |
| NV | 124,492.0 | 161,929.3 | 172,628.0 | 9.4 | 61,332.0 | 100,551.7 | 115,829.0 | 5.7 |
| NY | 644,715.0 | 844,950.8 | 900,759.0 | 7.9 | 352,393.0 | 570,080.1 | 655,963.0 | 5.2 |
| OH | 642,219.0 | 754,216.8 | 794,414.0 | 13.4 | 274,266.0 | 389,686.5 | 442,351.0 | 6.8 |
| OK | 174,338.0 | 221,415.3 | 234,194.0 | 9.3 | 84,162.0 | 133,728.4 | 152,656.0 | 5.5 |
| OR | 165,416.0 | 200,899.2 | 211,888.0 | 10.8 | 98,470.0 | 140,247.2 | 159,659.0 | 7.3 |
| PA | 605,891.0 | 725,076.9 | 764,881.0 | 11.7 | 299,051.0 | 428,097.5 | 486,670.0 | 6.8 |
| RI | 40,939.0 | 49,829.7 | 52,665.0 | 10.9 | 23,709.0 | 33,868.1 | 38,542.0 | 7.2 |
| SC | 248,450.0 | 301,009.6 | 318,559.0 | 12.0 | 114,117.0 | 168,906.6 | 192,296.0 | 6.6 |
| SD | 38,530.0 | 46,054.0 | 48,464.0 | 10.8 | 18,297.0 | 26,380.2 | 29,939.0 | 6.1 |
| TN | 376,781.0 | 440,573.1 | 464,611.0 | 14.2 | 153,721.0 | 218,299.7 | 248,167.0 | 6.9 |
| TX | 998,969.0 | 1,289,647.3 | 1,382,427.0 | 8.6 | 595,400.0 | 919,265.7 | 1,061,332.0 | 6.1 |
| UT | 45,906.0 | 64,038.4 | 67,865.0 | 4.7 | 32,631.0 | 54,761.9 | 62,805.0 | 4.0 |
| VA | 310,489.0 | 394,477.2 | 418,488.0 | 8.9 | 152,862.0 | 242,837.8 | 278,226.0 | 5.3 |
| VT | 26,589.0 | 31,638.0 | 33,190.0 | 9.5 | 15,252.0 | 21,347.7 | 24,177.0 | 6.3 |
| WA | 225,306.0 | 288,719.3 | 305,426.0 | 8.3 | 145,210.0 | 219,749.6 | 251,161.0 | 6.2 |
| WI | 248,506.0 | 298,032.1 | 313,913.0 | 11.1 | 127,493.0 | 182,376.5 | 207,161.0 | 6.6 |
| WV | 128,757.0 | 146,613.0 | 153,714.0 | 15.4 | 48,351.0 | 66,585.9 | 75,355.0 | 6.8 |
| WY | 27,529.0 | 32,751.6 | 34,494.0 | 11.3 | 14,177.0 | 19,958.9 | 22,683.0 | 6.7 |

Table A3 LCS Estimates Outliers, Census Tracts Where Estimates of LCS eligible persons Outnumber the Total Population

| **geoid** | **county** | **state_** | **state** | **current** | **former** | **total** | **population** | **percent** |
| --- | --- | --- | --- | --- | --- | --- | --- | --- |
| 12073001300 | 073 | 12 | FL | 1.830 | 1.650 | 3.480 | 1.500 | 2.320 |
| 17019000401 | 019 | 17 | IL | 1.910 | 1.780 | 3.690 | 2.900 | 1.272 |
| 26065980000 | 065 | 26 | MI | 1.880 | 1.870 | 3.750 | 3.000 | 1.250 |
| 36001000404 | 001 | 36 | NY | 4.850 | 4.300 | 9.150 | 7.400 | 1.236 |
| 15003009510 | 003 | 15 | HI | 2.860 | 2.550 | 5.410 | 1.600 | 3.381 |
| 42029980000 | 029 | 42 | PA | 0.940 | 0.850 | 1.790 | 1.500 | 1.193 |
| 06073005500 | 073 | 06 | CA | 0.940 | 0.900 | 1.840 | 1.000 | 1.840 |
| 22071980000 | 071 | 22 | LA | 3.790 | 3.490 | 7.280 | 6.600 | 1.103 |
| 34001983400 | 001 | 34 | NJ | 2.810 | 2.650 | 5.460 | 5.000 | 1.092 |
| 25001014100 | 001 | 25 | MA | 3.830 | 3.440 | 7.270 | 7.000 | 1.039 |
| 21067000801 | 067 | 21 | KY | 1.720 | 1.720 | 3.440 | 2.300 | 1.496 |
| 25023980300 | 023 | 25 | MA | 1.880 | 1.720 | 3.600 | 3.000 | 1.200 |
| 45013000400 | 013 | 45 | SC | 4.720 | 4.300 | 9.020 | 9.000 | 1.002 |
| 24003980000 | 003 | 24 | MD | 4.740 | 4.440 | 9.180 | 6.100 | 1.505 |
| 45091060600 | 091 | 45 | SC | 2.790 | 2.600 | 5.390 | 4.500 | 1.198 |
| 26073000703 | 073 | 26 | MI | 4.770 | 4.490 | 9.260 | 3.800 | 2.437 |
| 42119980800 | 119 | 42 | PA | 2.870 | 2.650 | 5.520 | 2.600 | 2.123 |
| 26083980100 | 083 | 26 | MI | 0.930 | 0.840 | 1.770 | 0.500 | 3.540 |
| 48041002100 | 041 | 48 | TX | 4.700 | 4.310 | 9.010 | 6.500 | 1.386 |
| 36109001200 | 109 | 36 | NY | 1.840 | 1.690 | 3.530 | 1.500 | 2.353 |
| 53053072909 | 053 | 53 | WA | 0.920 | 0.890 | 1.810 | 1.500 | 1.207 |
| 36029940100 | 029 | 36 | NY | 0.860 | 0.810 | 1.670 | 1.500 | 1.113 |
| 36061029700 | 061 | 36 | NY | 5.450 | 5.140 | 10.590 | 8.500 | 1.246 |
| 40109980004 | 109 | 40 | OK | 4.680 | 4.260 | 8.940 | 7.000 | 1.277 |
| 47125980100 | 125 | 47 | TN | 0.960 | 0.900 | 1.860 | 1.500 | 1.240 |

Table A4. Subpopulation Estimates and Percentages of PWS and PWFS

| Race / Ethnicity | Sex | Age Group | Estimate of PWS and Percentage of Total PWS | Estimate of PWFS and Percentage of Total PWFS |
| --- | --- | --- | --- | --- |
| American Indian | Female | 50-54 | 18,320.94 (0.11%) | 11,824.12 (0.11%) |
| American Indian | Female | 55-64 | 33,283.65 (0.21%) | 19,786.24 (0.19%) |
| American Indian | Female | 65-74 | 19,029.71 (0.12%) | 12,897.13 (0.12%) |
| American Indian | Female | 75-80 | 5,469.24 (0.03%) | 4,007.98 (0.04%) |
| American Indian | Male | 50-54 | 18,626.26 (0.12%) | 12,545.41 (0.12%) |
| American Indian | Male | 55-64 | 32,166.17 (0.20%) | 19,871.47 (0.19%) |
| American Indian | Male | 65-74 | 17,215.92 (0.11%) | 11,934.36 (0.12%) |
| American Indian | Male | 75-80 | 4,667.15 (0.03%) | 3,410.38 (0.03%) |
| Asian | Female | 50-54 | 83,770.68 (0.52%) | 57,283.79 (0.55%) |
| Asian | Female | 55-64 | 127,703.48 (0.79%) | 88,725.79 (0.86%) |
| Asian | Female | 65-74 | 75,499.89 (0.47%) | 69,484.35 (0.67%) |
| Asian | Female | 75-80 | 24,863.98 (0.15%) | 23,096.82 (0.22%) |
| Asian | Male | 50-54 | 81,598.93 (0.51%) | 59,163.86 (0.57%) |
| Asian | Male | 55-64 | 121,278.54 (0.75%) | 88,732.30 (0.86%) |
| Asian | Male | 65-74 | 70,329.42 (0.44%) | 64,385.11 (0.62%) |
| Asian | Male | 75-80 | 22,610.10 (0.14%) | 20,815.60 (0.20%) |
| Black | Female | 50-54 | 227,118.88 (1.41%) | 118,257.79 (1.14%) |
| Black | Female | 55-64 | 430,274.74 (2.67%) | 218,683.70 (2.12%) |
| Black | Female | 65-74 | 233,615.10 (1.45%) | 147,837.65 (1.43%) |
| Black | Female | 75-80 | 72,870.40 (0.45%) | 48,794.74 (0.47%) |
| Black | Male | 50-54 | 218,079.89 (1.35%) | 118,765.07 (1.15%) |
| Black | Male | 55-64 | 395,878.00 (2.46%) | 209,961.49 (2.03%) |
| Black | Male | 65-74 | 198,253.05 (1.23%) | 126,798.53 (1.23%) |
| Black | Male | 75-80 | 52,956.18 (0.33%) | 36,119.17 (0.35%) |
| Hispanic | Female | 50-54 | 216,824.70 (1.35%) | 146,124.76 (1.41%) |
| Hispanic | Female | 55-64 | 310,022.95 (1.93%) | 211,449.56 (2.05%) |
| Hispanic | Female | 65-74 | 155,642.25 (0.97%) | 134,524.91 (1.30%) |
| Hispanic | Female | 75-80 | 52,826.81 (0.33%) | 46,448.46 (0.45%) |
| Hispanic | Male | 50-54 | 243,736.90 (1.51%) | 167,225.70 (1.62%) |
| Hispanic | Male | 55-64 | 326,790.96 (2.03%) | 226,315.82 (2.19%) |
| Hispanic | Male | 65-74 | 150,210.97 (0.93%) | 127,064.64 (1.23%) |
| Hispanic | Male | 75-80 | 43,870.43 (0.27%) | 37,759.77 (0.37%) |
| Pacific Islander | Female | 50-54 | 3,262.63 (0.02%) | 2,392.17 (0.02%) |
| Pacific Islander | Female | 55-64 | 4,862.41 (0.03%) | 3,616.45 (0.03%) |
| Pacific Islander | Female | 65-74 | 2,824.74 (0.02%) | 2,556.14 (0.02%) |
| Pacific Islander | Female | 75-80 | 0,849.41 (0.01%) | 0,761.85 (0.01%) |
| Pacific Islander | Male | 50-54 | 3,314.32 (0.02%) | 2,600.33 (0.03%) |
| Pacific Islander | Male | 55-64 | 5,109.97 (0.03%) | 4,061.66 (0.04%) |
| Pacific Islander | Male | 65-74 | 2,651.22 (0.02%) | 2,446.81 (0.02%) |
| Pacific Islander | Male | 75-80 | 0,709.39 (0.00%) | 0,643.17 (0.01%) |
| White | Female | 50-54 | 1,164,190.45 (7.23%) | 685,252.53 (6.63%) |
| White | Female | 55-64 | 2,568,427.50 (15.95%) | 1,503,403.34 (14.55%) |
| White | Female | 65-74 | 1,663,258.76 (10.33%) | 1,158,179.89 (11.21%) |
| White | Female | 75-80 | 570,684.08 (3.54%) | 404,454.60 (3.91%) |
| White | Male | 50-54 | 1,248,252.62 (7.75%) | 766,164.19 (7.41%) |
| White | Male | 55-64 | 2,634,476.53 (16.36%) | 1,601,389.09 (15.50%) |
| White | Male | 65-74 | 1,643,226.54 (10.20%) | 1,148,273.99 (11.11%) |
| White | Male | 75-80 | 503,001.36 (3.12%) | 356,926.81 (3.45%) |

Figure A1. Distribution of BRFSS Prevalence Rates for Current Smoking and Quit Smoking, 2016-2020.
